# Global Gene Expression Analysis of Canine Osteosarcoma Stem Cells Reveals a Novel Role for COX-2 in Tumour Initiation

**DOI:** 10.1371/journal.pone.0083144

**Published:** 2014-01-08

**Authors:** Lisa Y. Pang, Emma L. Gatenby, Ayako Kamida, Bruce A. Whitelaw, Ted R. Hupp, David J. Argyle

**Affiliations:** 1 Royal (Dick) School of Veterinary Studies and Roslin Institute, The University of Edinburgh, Easter Bush, Midlothian, United Kingdom; 2 Edinburgh Cancer Research UK Centre, The University of Edinburgh, Edinburgh, United Kingdom; Colorado State University, United States of America

## Abstract

Osteosarcoma is the most common primary bone tumour of both children and dogs. It is an aggressive tumour in both species with a rapid clinical course leading ultimately to metastasis. In dogs and children distant metastasis occurs in >80% of individuals treated by surgery alone. Both canine and human osteosarcoma has been shown to contain a sub-population of cancer stem cells (CSCs), which may drive tumour growth, recurrence and metastasis, suggesting that naturally occurring canine osteosarcoma could act as a preclinical model for the human disease. Here we report the successful isolation of CSCs from primary canine osteosarcoma, as well as established cell lines. We show that these cells can form tumourspheres, and demonstrate relative resistance to chemotherapy. We demonstrate similar results for the human osteosarcma cell lines, U2OS and SAOS2. Utilizing the Affymetrix canine microarray, we are able to definitively show that there are significant differences in global gene expression profiles of isolated osteosarcoma stem cells and the daughter adherent cells. We identified 13,221 significant differences (p = 0.05), and significantly, COX-2 was expressed 141-fold more in CSC spheres than daughter adherent cells. To study the role of COX-2 expression in CSCs we utilized the COX-2 inhibitors meloxicam and mavacoxib. We found that COX-2 inhibition had no effect on CSC growth, or resistance to chemotherapy. However inhibition of COX-2 in daughter cells prevented sphere formation, indicating a potential significant role for COX-2 in tumour initiation.

## Introduction

Osteosarcoma is the most common bone tumor in children and adolescents comprising 20% of all bone tumors and about 5% of pediatric tumors overall [1], [2]. The highest incidence of osteosarcoma is in the second decade of life, suggesting a relationship between bone growth and tumor development [3], [4]. Significant improvements in patient survival rates have been achieved through multimodal therapeutic approaches combining high-dose chemotherapy and surgical resection [5]. However, despite these advances, the overall relapse free-survival rate over 5-years has remained at approximately 65% to 75% and the intensification of chemotherapy regimens has not improved survival [6], [7]. Like the situation in children, osteosarcoma is the most commonly diagnosed primary bone tumour of dogs [8]. It generally occurs on the limbs of middle-aged to older, large breed dogs, with the distal radius and proximal humerus as common locations [8]. These neoplasms are highly malignant with aggressive local effects and a high risk of metastasis to the lungs. In dogs, 1-year survival times are <20% despite surgery and chemotherapy [8].

In recent years the traditional stochastic model of cancer development has been challenged by a new model, which implicates cancer stem cells as the subpopulation of cancer cells that maintains the malignant phenotype [9]. These cancer stem cells (CSCs) share several characteristics with embryonic and somatic stem cells including self-renewal and differentiation abilities, and represent a small fraction of the cellular population of the tumour. The role of CSCs was initially established in leukaemia, and more recently in solid tumours including melanomas [10], [11], glioblastomas [12] and epithelial cancers [13], [14], [15], [16], [17]. Increasing evidence has implicated CSCs in tumorigenesis and response to treatment of many tumour types [6], [18], [19], [20]. Significantly, the resistance of these cells to conventional chemotherapeutic regimes suggests that CSCs play a major role in drug resistance and treatment failure [21].

Osteosarcoma CSCs have been identified in humans and dogs suggesting that these cells may be responsible for treatment failure in this disease [22], [23], [24], [25], [26]. The fact that current therapeutic strategies have not improved survival times for either species in recent years obviates the explicit need for osteosarcoma CSCs to be characterized to identify therapeutic targets [19]. As both canine and human osteosarcoma has been shown to contain a subpopulation of CSCs, which may drive tumour growth, recurrence and metastasis, this represents an opportunity to develop a natural pre-clinical model of a human disease in dogs that has greater relevance than current induced or xenograft rodent models [9], [27].

Previously we have identified CSCs in canine osteosarcoma cell lines [22]. In this present study we isolated CSCs from a primary osteosarcoma patient presented for treatment at the University of Edinburgh Veterinary Cancer Centre. We have identified a subpopulation of cells with stem-like properties in canine osteosarcoma that is relatively resistant to conventional chemotherapy. Global transcriptional analysis and comparison with parental cells identified COX-2 expression to be significantly increased in this population. Interestingly, several histological studies of human and canine osteosarcoma implicate COX-2 in tumour growth and progression, underpinning therapeutic strategies utilizing COX-2 inhibitors. We find that COX-2 inhibition had no effect on CSC growth, or resistance to chemotherapy. However inhibition of COX-2 in daughter cells prevented sphere formation. Similar findings were also observed in sphere cells derived from human osteosarcoma cell lines. Based on these observations, we believe that CSCs play a critical role in determining the response of osteosarcoma patients to therapy and COX-2 may play a role in tumour formation and maintenance. The similarities observed in canine and human cells underpin the dog as a potential pre-clinical model of osteosarcoma therapeutics.

## Results

### Osteosarcoma cells contain a subpopulation of cells with stem cell characteristics

**Figure 1:**
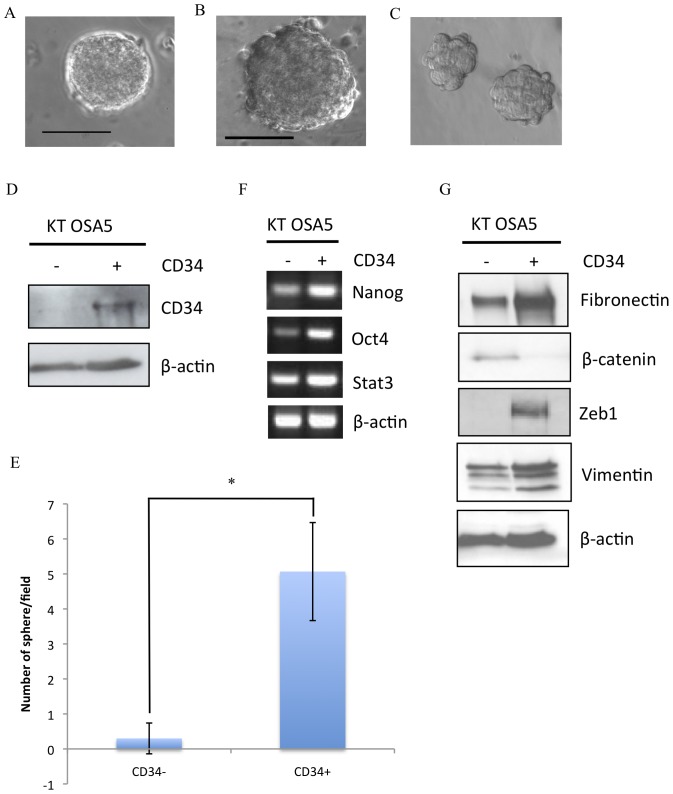
Characterisation of osteosarcoma stem cells. Spheres can be isolated from canine KTOSA5 cells (A), the human U2OS cell line (B), human SAOS2 cell line (C). A small population of CD34+ cells can be isolated from the KTOSA5 cell line by magnetic cell sorting. Only CD34+ cells express CD34 protein (D) and only CD34+ cells could form spheres (E). Data are representative of three independent experiments (*p<0.001). Reverse transcriptase (RT)-PCR analysis of *Nanog*, *Oct4*, *STAT3*, and *β-actin* gene expression levels (F). Western blots analysis of Fibronectin, β-catenin, Zeb1, and Vimentin, with β-actin as a loading control (G).

Previous studies have shown that CSCs derived from a variety of human tumours form spheroid colonies in defined serum free culture that favors the proliferation of undifferentiated cells [22]. Here, cells isolated from a primary canine osteosarcoma, KTOSA5, and human osteosarcoma cell lines, U2OS and SAOS2, were seeded as single cells at low-density into suspension cultures in serum-free growth factor supplemented media. After 5–7 days tumourspheres were clearly visible (Figure 1A, B and C, respectively). To determine whether tumourspheres can be expanded *in vitro*, spheres were dissociated into single cell suspensions and passaged multiple times in a long-term sphere-forming assay. These cells repeatedly formed tumourspheres for up to five subsequent passages when plated under the stated culture conditions and in the absence of attachment.

CD34 is a cell surface marker of hematopoietic stem cells [28], adipose derived stem cells [29] and cancer stem cells of skin cancer [30], colorectal adenocarcinoma [31], and gastrointestinal stromal tumours [32], [33]. CD34 expression is also primarily observed in mesenchymal tumours [34], [35]. Here we utilized CD34, to isolated CD34+ cells from the KTOSA5 cell line by magnetic cell sorting. The mean (± SD) number of CD34+ cells was 1.365%±0.34% (*n* = 8, where *n* is the number of times the experiment has been replicated). Western blot analysis confirmed that CD34 expression was confined to the CD34+ cell population (Figure 1D). Significantly, only CD34+ cells could form spheres when seeded in serum-free media (Figure 1E).

To further characterise CD34+ cells as a primitive subpopulation of KTOSA5 cells, we examined the expression of embryonic stem cell markers *Oct4*, *Nanog* and *STAT3*. Oct4 and Nanog are transcriptional determinants essential for self-renewal and maintenance of the undifferentiated state [36]. Here we show that *Oct4*, *Nanog*, and *STAT3* are expressed at a higher level in CD34+ compared to CD34- cells (Figure 1F).

We have previously shown that CSCs derived from a canine mammary carcinoma cell line have a mesenchymal phenotype [37]. Here we show a similar expression pattern for KTOSA5 cells, whereby expression of β-catenin was significantly decreased, and that of Fibronectin, Zeb1 and Vimentin was significantly increased in CD34+ cells compared to CD34- cells (Figure 1G). Thus the canine osteosarcoma cell line, KTOSA5, contains a subpopulation of cells that can survive in the absence of attachment; forms tumourspheres that can be expanded *in vitro*; expresses embryonic stem cell makers, which may be required for maintaining these cells in a primitive state; and expresses a mesenchymal phenotype.

### Osteosarcoma stem cells exhibit greater resistance to chemotherapy

**Figure 2:**
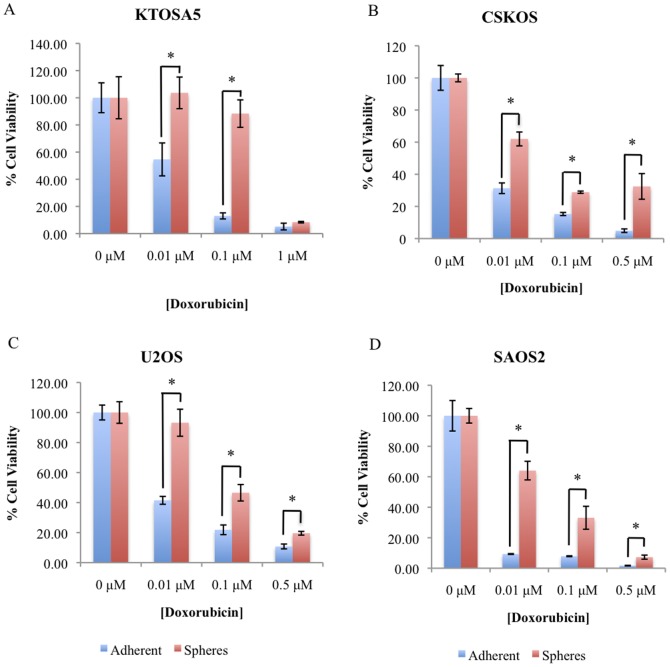
Cancer stem cells are resistant to the cytotoxic effects of doxorubicin. Spheres were isolated from the canine osteosarcoma cell lines; KTOSA5 (A) and CSKOS (B), and the human osteosarcoma cell lines U2OS (C) and SAOS2 (D). Spheres and adherent cells were treated with the indicated doses of doxorubicin and cell viability was assayed 48 hr after treatment (* *p*<0.005).

**Figure 3:**
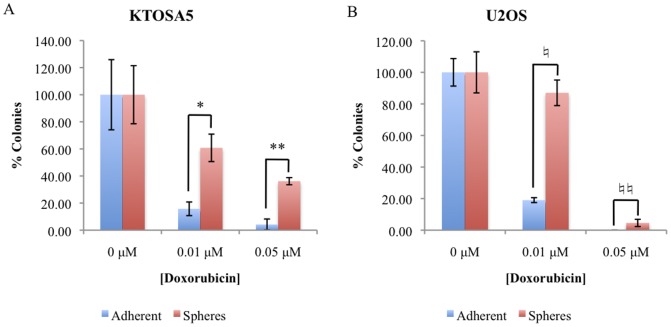
Spheres are resistant to replicative cell death after doxorubicin treatment. Colony forming ability after doxorubicin treatment was determined in KTOSA5 cells (* *p* = 0.008; ** *p*<0.001) (A) and U2OS cells (♮ *p* = 0.008; ♮♮ *p*<0.001) (B).

To determine whether tumourspheres cells preferentially survive after treatment with chemotherapeutic agents, tumourspheres derived from the canine osteosarcoma cell lines KTOSA5 and CSKOS, and from human osteosarcoma cell lines U2OS and SAOS2, were dissociated into single cells and treated with increasing concentrations of the chemotherapeutic drug, doxorubicin. Doxorubicin is an anti-tumour antibiotic DNA damaging agent and is commonly used in veterinary and human cancer chemotherapy protocols. We used doses of doxorubicin in cell culture experiments that correlate to concentrations that can be achieved *in vivo*. Cell viability was assayed 72 hours after treatment. Cells from tumourspheres demonstrated a significantly increased resistance to the cytotoxic effect of doxorubicin compared to parental adherent cells (Figure 2A, B, C, and D respectively). In addition, we compared the colony forming ability of disassociated spheres and adherent cells from the cell lines, KTOSA5 and U2OS, after doxorubicin treatment (Figure 3A and B). Both KTOSA5 and U2OS spheres are resistant to doxorubicin induced replicative cell death compared to adherent cells. Both canine and human osteosarcoma spheres are resistant to the therapeutic dose of DNA damaging agents *in vitro*, and therefore in a physiological setting may contribute to tumour repopulation.

### Tumourspheres increased invasiveness and tumourigenicity

**Figure 4:**
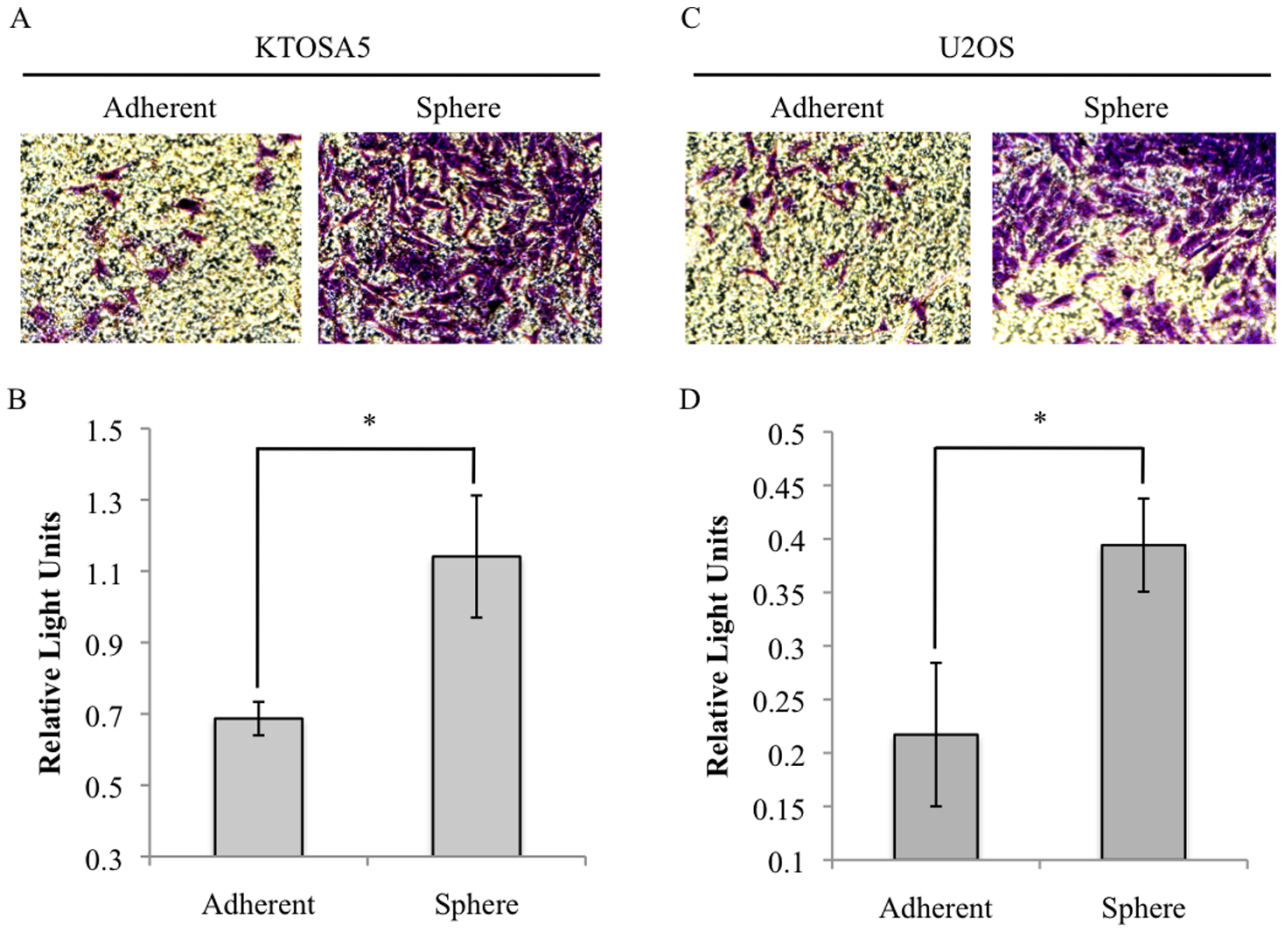
Cancer stem cells show an increased invasive potential *in vitro*. Invasive ability of KTOSA5 (A, B) and U2OS (C, D) spheres and adherent cells was analysed using a collagen based invasion assay. Invading cells were quantified by measuring the optical density at 560 nm. * *p*<0.005.

The metastatic process involves migration from the tumour microenvironment and subsequent invasion and attachment at a secondary site within the body. Here, the invasive capacity of cells dissociated from tumourspheres and matched adherent cells, was determined using a Boyden chamber assay. KTOSA5 tumourspheres displayed a significantly greater invasive potential compared to adherent cells (Figure 4A, *p*<0.005). Similar results were obtained for U2OS cells (Figure 4B, *p*<0.005). This data is consistent with the hypothesis that cancer stem cells contribute to invasion and migration of the tumour.

**Figure 5:**
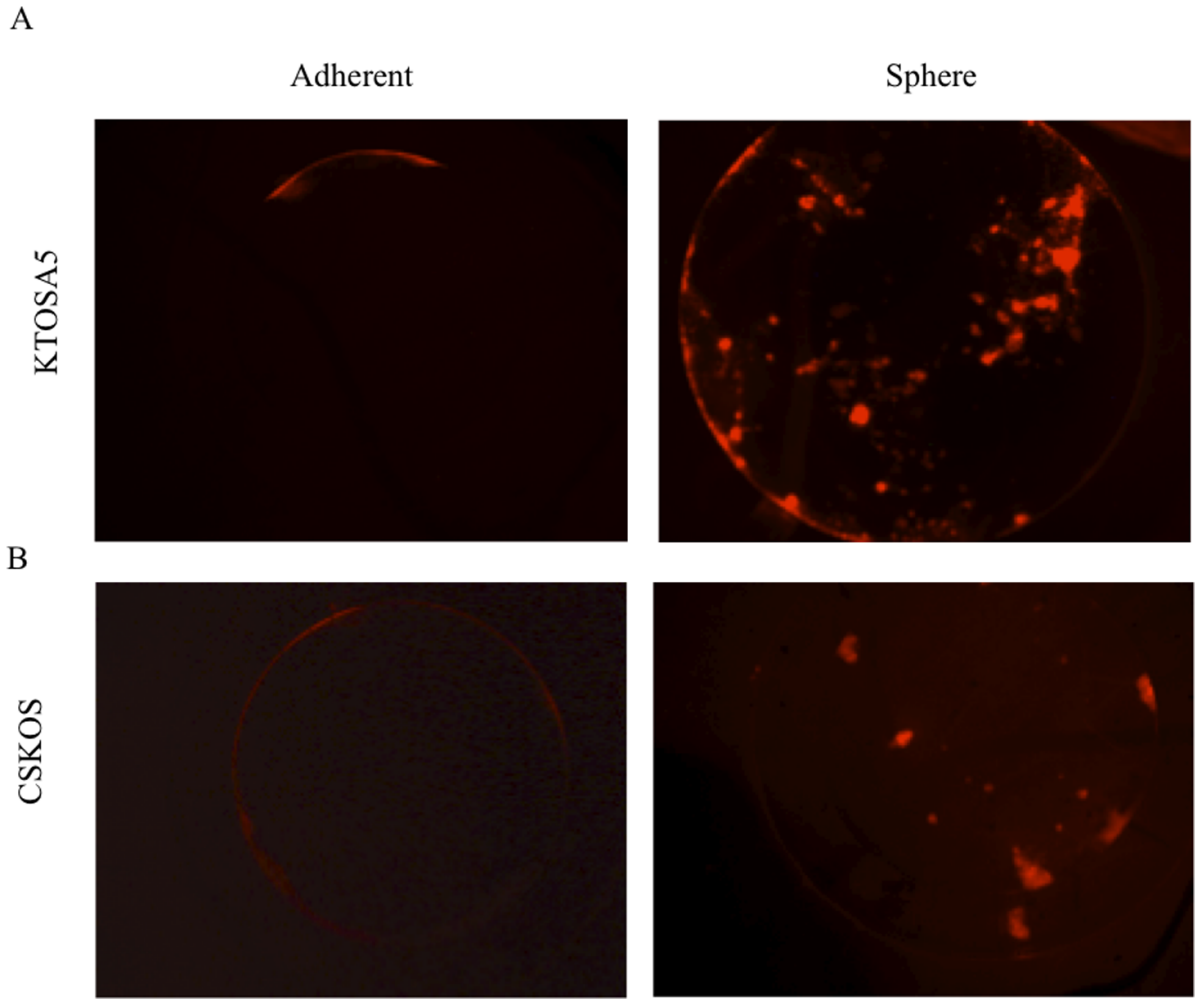
Osteosarcoma spheres are enriched for higher tumourigenicity *in vivo*. Disassociated spheres and adherent, of KTOSA5 cells (A) and CSKOS cells (B), were inoculated directly onto the chorioallantoic membrane of a chicken embryo at day 7 of development. All cells were fluorescently labelled and imaged 5 days after inoculation.

To evaluate tumourgenicity of the canine osteosarcoma cell lines, KTOSA5 and CSKOS, we utilised the chicken embryo chorioallantoic membrane (CAM) model. Chicken embryos were inoculated with fluorescently labelled dissociated spheres or adherent cells, directly on to their CAM, at day 7 of development. Five days after tumour cell inoculation the formation of 3-dimensional tumours became apparent in 100% of membranes inoculated with dissociated spheres but not adherent cells. These micro-tumours were visualized under the fluorescence microscope; sphere cells were brightly fluorescent and had radiated out from the 3-dimentional tumour growths, invading the surrounding blood vessels of the CAM. In contrast, adherent cells were localised to the initial site of inoculation and weakly fluorescent, possibly indicating that these cells were dying and unable to establish growth. Similar results were obtained in both KTOSA5 and CSKOS cell lines (Figure 5A and B, respectively). Thus, spheres have greater *in vivo* tumourigenic capacity than adherent cells.

### Analysis of gene expression

**Figure 6:**
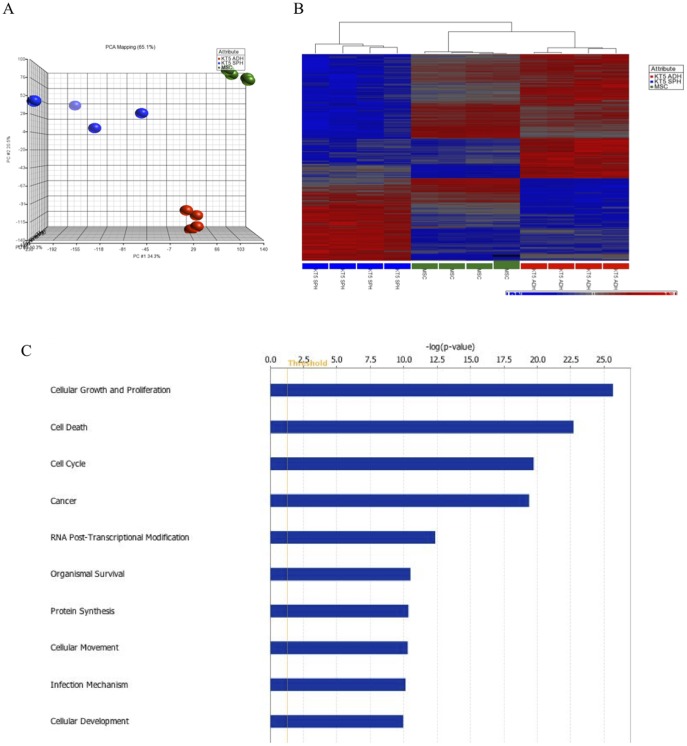
Gene expression analysis of canine osteosarcoma stem cells. A three-dimensional representation of a principle component analysis of expression microarray data derived from KTOSA5 adherent cells, spheres and mesenchymal stem cells (MSC) (A). Heirarchial clustering analysis of the expression data (cut off p-value of 0.005). Expression values are represented by colours: blue squares represent low-expressed genes, red squares represent high-expressed genes (B). Biological process analysis of differentially expressed genes in KTOSA5 spheres compared to adherent cells (FDR = 0.005) (C).

We performed gene expression profiling of KTOSA5 spheres using the Affymetrix GeneChip® Canine 2.0 Array. Cancer stem cells, represented by sphere cells, differentially expressed (i.e. up- or down regulated >2-fold with a false discovery rate (FDR) of 0.05) 13,221 genes compared to adherent cells. As a control, KTOSA5 spheres were also compared to canine mesenchymal stem cells (MSCs) [38]. Here there were 7,542 significant differences (FDR of 0.05), indicating that osteosarcoma stem cells are more similar to mesenchymal stem cells than the bulk adherent cells from which they were derived. To obtain a manageable number of gene differences, the FDR was decreased to 0.005. Under these parameters, 5,685 genes were differentially expressed in spheres compared to adherent cells. Principle component analysis shows a distinct separation of the three cell populations (Figure 6A) and the heatmap shows that CSCs cluster more closely with MSCs than adherent cells (Figure 6B). Further pathway analysis showed that the differential expression profile of spheres encompassed genes involved in a variety of biological processes and diseases including cell growth, proliferation, development, cell cycle regulation, apoptosis, protein synthesis, and cell movement (Figure 6C). Significantly, cancer was the top disease identified in the analysis (Table 1), indicating that gene expression profiles associated with cancer are more prevalent in the cancer stem cell population than the adherent cells. The top ten upregulated genes in CSCs compared to adherent cells are shown in Table 2.

**Table 1:** Top biological functions of differentially expressed genes in KTOSA5 spheres compared to adherent cells (FDR = 0.005).

	p-value	Number of Molecules
**Diseases and Disorders**		
Cancer	4.16E-20–4.95E-04	875
Infection Mechanism	7.19E-11–5.19E-04	359
Gastrointestinal Disease	2.08E-10–5.17E-04	377
Infectious Disease	2.87E-10–1.41E-04	345
Neurological Disease	4.56E-10–5.19E-04	827
**Molecular & Cellular Functions**		
Cellular Growth & Proliferation	2.20E-26–4.98E-04	731
Cell Death	1.99E-23–5.19E-04	705
Cell Cycle	1.87E-20–5.19E-04	364
RNA Post-translational Modification	4.35E-13–5.19E-04	98
Protein Synthesis	4.45E-11–4.11E-04	167
**Physiological System Development & Function**		
Organismal Survival	3.10E-11–1.04E-10	240
Organismal Development	3.05E-08–3.89E-04	299
Skeletal & Muscular System Development	3.60E-08–4.11E-04	90
Tumour Morphology	7.59E-07–2.11E-04	69
Cardiovascular System Development	8.29E-07–4.08E-04	137

**Table 2:** Top ten upregulated genes in KTOSA5 spheres compared to adherent cells (FDR = 0.005).

Gene Symbol	Gene Name	Accession Number	Gene Ontology	Fold Change
ALDH3A1	Aldehyde Dehydrogenase 3 Family, Member A1	E2RB52	Aldehyde metabolic process	177.26
PTGS2	Cycloxygenase 2	Q8SPQ9	Cell proliferation	141.31
PDK4	Pyruvate dehydrogenase kinase	E2RKY0	Protein phosphorylation	120.81
SNCG	Synuclein gamma	F1Q2N7	Unknown	79.19
IL6	Interleukin-6	P41323	Immune response	60.85
PTGER2	Prostaglandin E2 receptor EP2 subtype	Q9XT82	Signal transduction	46.10
RGS1	Regulator of G-Protein Signaling	F6XTL6	Termination of G-protein coupled receptor signalling	31.10
CXCL14	Chemokine (C-X-C motif) ligand 14	E2RCZ4	Immune response	30.59
SERPINB2	Serpin Peptidase inhibitor, Clade B, member 2	E2R079	Serine-type endopeptidase	30.57
CCL24	C-C motif chemokine 24	Q68Y68	Immune response	29.49

### COX-2 expression is elevated in cancer stem cells

**Figure 7:**
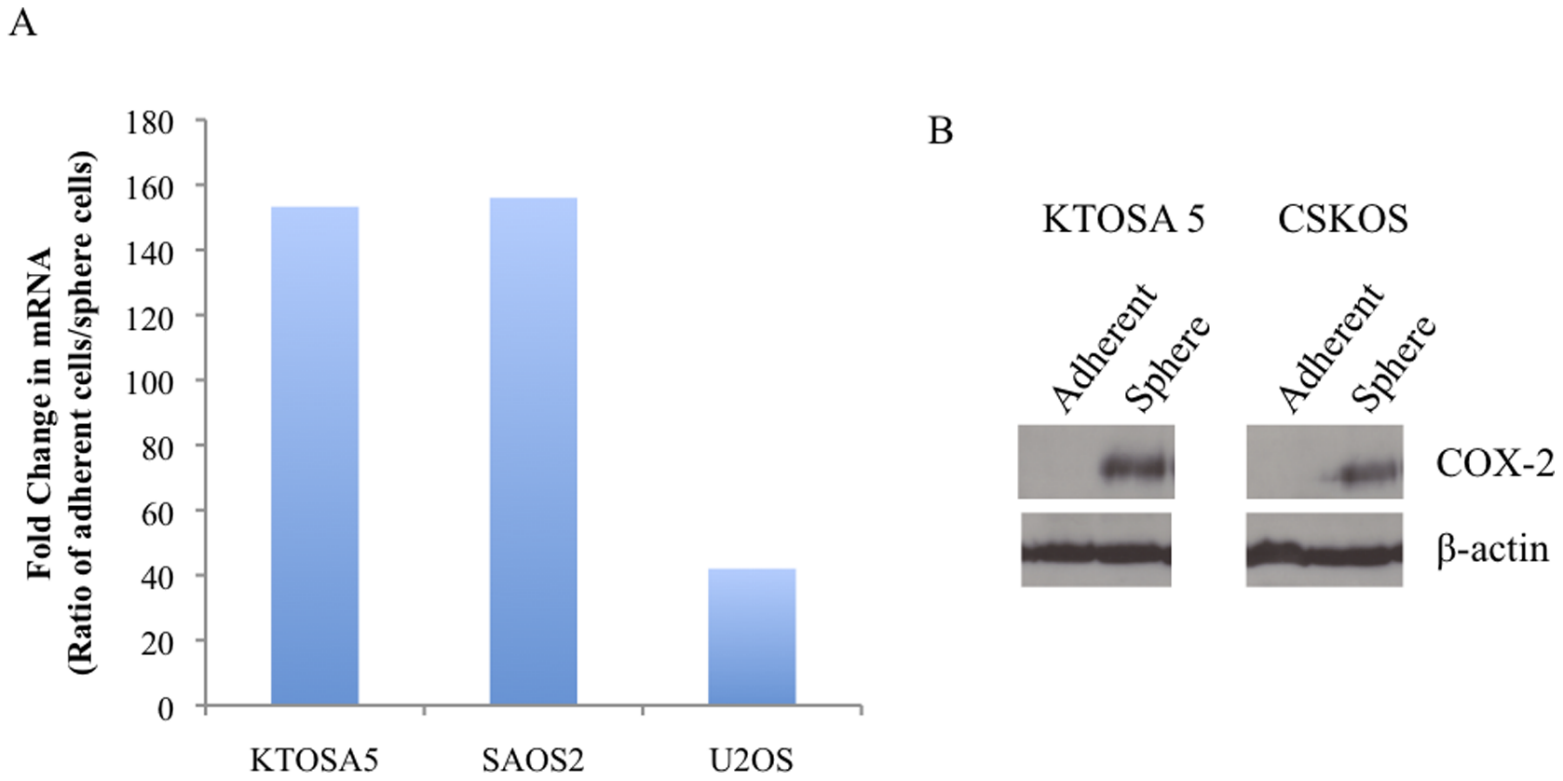
Cancer stem cells express a higher level of COX-2. Validation of microarray with qRT-PCR (A). Expression of COX-2 protein (B).

The microarray analysis identified COX-2 expression as being 141-fold up-regulated in KTOSA5 spheres compared to adherent cells. We confirmed this by qRT-PCR, and showed that COX-2 expression is up-regulated 153-fold in KTOSA5 spheres; 156-fold in SAOS2 spheres; and 42-fold in U2OS cells (Figure 7A). We also confirmed that COX-2 is elevated at the protein level in KTOSA and CSKOS spheres compared to adherent cells (Figure 7B).

### COX-2 has no effect cell viability, colony forming ability or chemosensitivity of cancer stem cells

**Figure 8:**
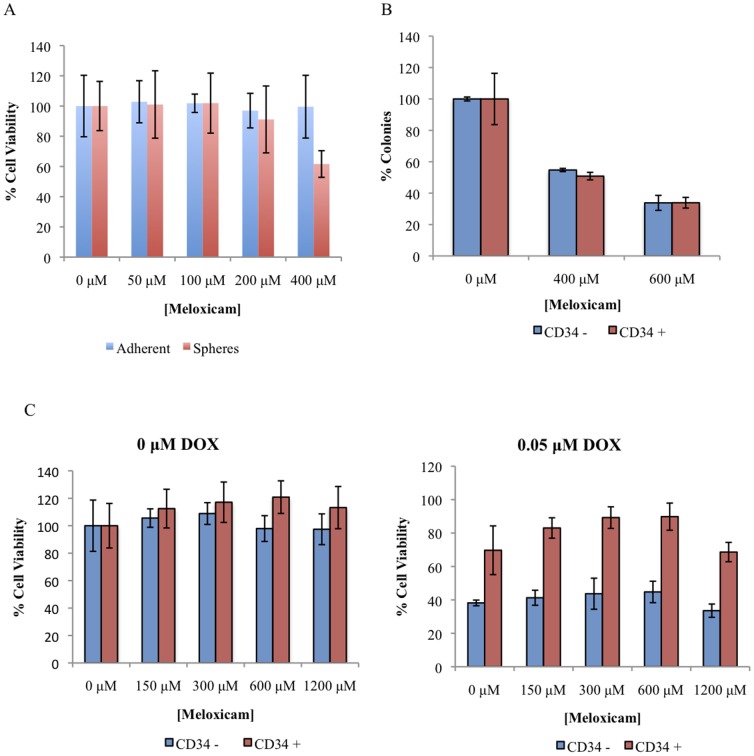
COX-2 inhibition has no effect on cell viablilty or chemo-resistance of cancer stem cells. Dissociated KTOSA5 spheres and adherent cells (A), and CD34 sorted KTOSA5 cells (B) were treated with the indicated doses of meloxicam and cell viability was assayed 72 hr after treatment. KTOSA5 CD34- and CD34+ cells were treated with both indicated doses of meloxicam and 0.05 µM doxorubicin, cell viability was assayed 72 hr after treatment.

To elucidate the role of COX-2 in cancer stem cell biology, we used the COX-2 inhibitor meloxicam. Increasing doses of meloxicam had no significant effect on the cell viability of KTOSA5 cells, and there was no difference between spheres and adherent cells (Figure 8A). A colony formation assay showed that COX-2 inhibition by meloxicam could decrease long-term cell survival, in a dose-dependent fashion, using high doses of the drug. As before there was no difference between the CD34+ cells, representing the cancer stem cell population, and the CD34- cells (Figure 8B). COX-2 inhibition by meloxicam also had no effect on the resistance of cancer stem cells to doxorubicin treatment (Figure 8C).

### COX-2 is required for tumoursphere formation

**Figure 9:**
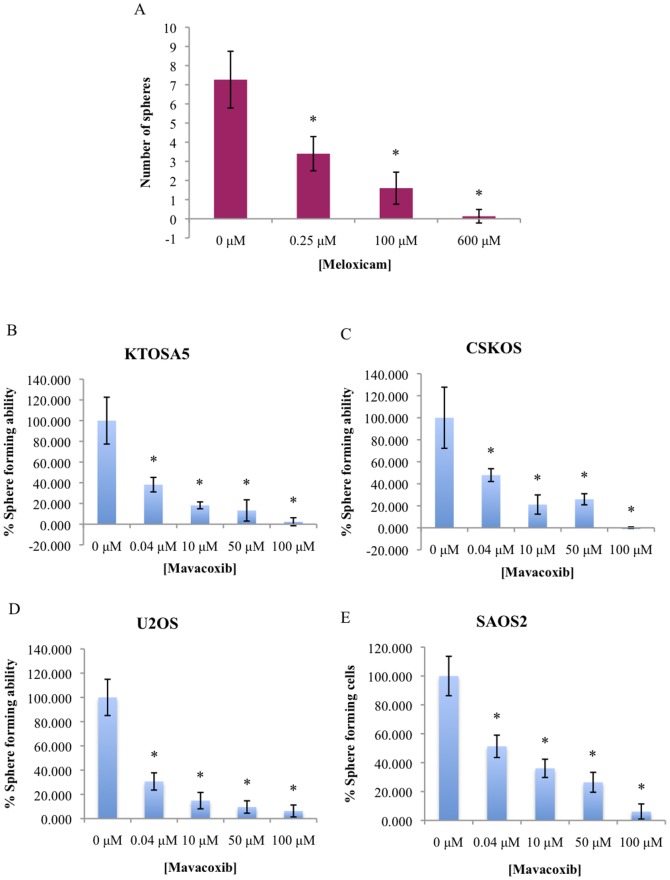
COX-2 inhibition suppresses sphere forming ability. KTOSA cells were pre-treated for 24 hr with the indicated doses of meloxicam prior to assaying for sphere forming ability (* *p*<0.001) (A). KTOSA5 (B), CSKOS (C), U2OS (D) and SAOS2 (E) cells were pre-treated for 24 hr with the long-acting COX-2 inhibitor mavacoxib prior to assaying for sphere forming ability (* *p*<0.001).

To determine if COX-2 has an effect on the ability of cancer cells to form spheres, KTOSA5 adherent cells were pretreated with either 0 µM, 0.25 µM, 100 µM, or 600 µM meloxicam and seeded at 6000 cells per well in serum-free sphere forming media. After 7 days the number of spheres per field were counted. There were significantly less spheres in the meloxicam treated plates compared to the 0 µM vehicle (DMSO) control treated cells (Figure 9A, *p*<0.01). To confirm this result with a different COX-2 inhibitor and in additional cell lines, we treated KTOSA5, CSKOS, U2OS and SAOS2 adherent cells with increasing doses (0 µM, 0.04 µM, 10 µM, 50 µM, 100 µM) of the long-acting COX-2 inhibitor, mavacoxib and seeded the cells appropriately for a sphere-forming assay (Figure 9B–E, *p*<0.001). As before, there was a striking decrease in the number of spheres formed in all cell lines tested, which is dose-dependent on COX-2 inhibition. This data indicates that COX-2 plays a central role in sphere forming ability.

## Discussion

The identification of CSCs in osteosarcoma has enormous implications for therapeutic development. In human osteosarcoma survival rates have remained static for the past 20 years, development of drug resistance being a major feature of treatment failure [6], [7]. In the dog, a similar situation prevails with 1 year survival times being well below 20% [8]. The resistance of CSCs to conventional cytotoxic therapy makes it a prerequisite to characterize these cells in terms of potential therapeutic targets [9]. Previously we have demonstrated the enrichment of these cells in established canine osteosarcoma cell lines [22]. In this study we reinforced the hypothesis of the stem cell basis for osteosarcoma, by achieving isolation of such a population from clinical samples.

A fundamental property of CSCs is their ability to self-renew [9]. The sphere-forming capacity of KTOSA5 cells that was observed following five serial passages under selective culture conditions demonstrated the presence of a self-renewing cell population. Furthermore, when inoculated on to the chorioallantoic membrane of chicken embryos, spheres were much better at initiating and establishing tumour growth than adherent cells. Therefore canine osteosarcoma stem cells, in comparison with daughter adherent cells, express embryonic stem cell markers; can self-renew; are resistant to the cytotoxic effects of chemotherapeutic drugs; are more invasive *in vitro*; and are more tumourigenic *in vivo*. Significantly, our microarray data showed vast differences in the gene expression profiles of KTOSA5 spheres and adherent cells, with 13,221 significant differences. Data mining for biologically relevant processes identified that overexpressed genes in spheres are associated with cancer, cell growth and proliferation, cell cycle regulation, and organismal survival. Previous studies have suggested that CSCs derived from bone sarcomas arise from mesenchymal stem cells (MSC) [39]. Classically MSCs lack expression of CD34 [40], [41], but here we show that a small subpopulation of CD34+ cells can be isolated from KTOSA5 cells with characteristics of cancer stem cells. Furthermore, we compared global gene expression of canine osteosarcoma CSC to canine MSCs. Although CSCs were more similar to MSCs than adherent cells, there were still 7,542 significant differences. Pathway analysis showed that genes associated with cancer, growth regulation and cell cycle regulation are still differentially expressed and represent fundamental differences between CSCs and MSCs.

To identify potential therapeutic targets in the CSC population, a list of the most differentially expressed genes was compiled. The top gene upregulated, by 177 fold, in spheres compared to adherent cells is ALDH3A1. This gene is a member of the aldehyde dehydrogenase family, which catalyses the oxidation of aldehydes and serves a detoxifying role [42]. Previous studies have shown that ALDH1A1 activity is a marker of normal hematopoietic stem cells and of CSC enriched populations of multiple human malignancies including breast, colon, pancreas, lung and liver [43], [44]. Therefore in canine osteosarcoma cells, ALDH3A1 expression may contribute to the resistance of the CSC pool to chemotherapeutic drugs.

COX-2 expression is also significantly elevated, by 141-fold, in the CSC pool. This finding has important biological and therapeutic implications. Advances in our understanding of the pathways involved in cancer related inflammation could enable the development of synergistic therapies that target the tumour promoting effects of the inflammatory microenvironment [45]. Cyclooxygenase-2 (COX-2) is an inducible prostaglandin synthetase with a key role in regulating inflammation [46]. There is now mounting evidence to suggest that COX-2 and prostaglandins (PGs) play a vital role in various aspects of carcinogenesis including the promotion of angiogenesis and the down-regulation of apoptosis [46], [47], [48], [49]. Previous studies have shown that COX-2 is not expressed in normal bone in dogs [50] whereas 77% of 44 osteosarcomas were found to be positive for COX-2 expression [51]. A more recent study confirms this data [52]. COX-2 expression has also been established as a marker in human osteosarcoma, and COX-2 inhibition has been suggested as a possible way of improving therapeutic outcome [53], [54], [55]. Given the postulated links between COX-2 and tumour development, we aimed to investigate the antitumor activity of COX-2 inhibitors in osteosarcoma cell lines and derived CSCs. Previous studies have examined the expression of key inflammatory mediators to determine whether COX-2 inhibition can block the induction of inflammation in these cells, and have shown that COX-2 expression correlates with tumour grade and survival [55]. Interestingly, the data suggests COX-2 overexpression in the primary tumour correlates with the occurrence of distant metastasis in patients with osteosarcoma and also may affect post-metastatic survival [55]. Similarly, in a model of breast cancer metastasis to the bone, COX-2 plays a key role in the development of osteolytic bone metastasis [56]. In breast cancer stem cells, isolated from the primary tumours of HER2/Neu transgenic mice, COX-2 expression was upregulated 30-fold in spheres compared to adherent cells, and constituted part of an eight-gene signature that correlated with breast cancer patient survival [57]. Furthermore, transfection of COX-2 into the ER-positive breast cancer cell line, MCF7, increased the ability of MCF7 cells to grow as tumourspheres [58]. However, to date there have been no such studies relating to the effects of COX-2 on osteosarcoma stem cells. Having demonstrated that COX-2 expression is significantly elevated in the CSC population we hypothesized that COX-2 inhibition could serve to target this population as part of an overall therapeutic strategy. Initially, we investigated whether COX-2 inhibition would inhibit cell viability. Increasing doses of meloxicam had no significant affect on the cell viability of CSCs or adherent cells. However, high doses of meloxicam, 400 µM and 600 µM, could decrease long-term cell survival of both CSCs and adherent cells. These results are consistent with a previous study showing that growth inhibition of the canine osteosarcoma cell line, D17, was seen after 48 hr treatment with 400 µM and 600 µM meloxicam [59]. We also show that COX-2 inhibition by meloxicam did not improve sensitivity of CSCs to conventional chemotherapeutic drugs. However, although we demonstrate that COX-2 inhibition had no effect of CSC viability or chemo-resistance, there was a significant effect on the sphere-forming capacity of daughter cells. We consistently showed that COX-2 inhibition by either meloxicam or mavacoxib induced a dose-dependent decrease in sphere forming ability in all canine and human osteosarcoma cell lines tested. Importantly, the lowest doses we tested, 0.04 µM mavacoxib and 0.25 µM meloxicam, can be achieved *in vivo*. In dogs the mean plasma concentration of mavacoxib is 1.35–2.88 µM on day 14 when the drug is administered at 2 mg/ml on day 0, day 14 then monthly [60]. Similarly, the mean plasma concentration in dogs subcutaneously administered with a single dose of meloxicam at 0.2 mg/kg after 24 hr is 1.32 µM–2.09 µM [61]. This signifies that the doses we have tested are clinically relevant.

Our data is consistent with a previous study in which mouse embryonic stem cells lacking functional COX-2 have a normal growth rate and differentiation potential but are profoundly compromised in their ability to form aggressive teratocarcinomas *in vivo* [62]. Taken together this data indicates that COX-2 plays a major role in tumour initiation. Further experimentation is required to determine if inhibition of COX-2 can prevent metastasis, and to evaluate the potential of COX-2 inhibitors as chemopreventative agents of osteosarcoma.

## Materials and Methods

### Cell Culture and Sphere Formation

Canine osteosarcoma cells; KTOSA5 and CSKOS were grown in Dulbecco's modified Eagle's medium (DMEM) (Invitrogen, Paisley, UK) supplemented with 10% fetal bovine serum and 100 µg/ml streptomycin (Invitrogen, Paisley, UK). The KTOSA5 cell line was derived from an osteosarcoma affecting the right hind limb of an 8-year-old, chemotherapy naïve, female entire Rottweiler (approved by the University of Edinburgh Veterinary Ethical Review Committee). The CSKOS cell line (previously called KOS-003) was characterized by Hong et al., 2010 [63] and was a kind gift from Chand Khanna, NIH. Canine Mesenchymal Stem Cells (MSCs) were derived from canine bone marrow as described by Hodgekiss-Geere et al., 2012 [38]. Briefly, primary canine MSCs were isolated from bone marrow aspirates and characterized using marker expression and morphology (expression of CD44 and STRO-1, but not CD34 or CD45). Human osteosarcoma cells; U2OS and SAOS2 were grown in DMEM (Invitrogen, Paisley, UK) supplemented with 10% fetal bovine serum and 100 µg/ml streptomycin.

For anchorage-independent culture, osteosarcoma cells were plated as single cells in ultralow attachment 6-well plates (Corning, CA, USA) at low cell density (1.5×10^4^ cells/ml). Cells were grown in serum-free conditional medium, which contained William's E Medium with GlutaMAX supplemented with putrescine (100 µM), sodium selenite (30 nM), transferring (25 µg/ml), insulin (20 µg/ml) (Sigma Biochemicals, Dorset, UK), human recombinant bFGF (10 ng/ml) and EGF (10 ng/ml) (Peprotech, NJ, USA). Additional growth factors (100 µg/ml) were added to the media every other day. All cell cultures were maintained at 37°C in a humidified CO_2_ incubator.

### Magnetic cell sorting

Cells were labelled with CD34 microbeads and sorted using the Miltenyi Biotec CD34 cell isolation kit according to the manufacturer's protocol (Miltenyi Biotec, Surrey, UK). Briefly, cells were resuspended in 300 µl PBS solution (pH 7.2, 0.5% BSA, 2 mM EDTA) per 10^8^ cells. Then blocking reagent FcR (100 µl/10^8^ cells; Miltenyi Biotec, Surrey, UK) and CD34 microbeads (100 µl/10^8^ cells) were added and mixed at 4°C for 30 minutes with rotation. Cells were washed in 20× volume with PBS solution. The pellet was resuspended in 500 µl PBS solution and added to a pre-washed magnetic separation (LS) column on the magnetic holder. The column was washed four times and the cells were collected as the negative fraction. The column was removed from the magnetic holder and the positive fraction was collected.

### Sphere forming efficiency

The sphere forming ability of CD34 sorted cells, and cells treated with the indicated dose of COX-2 inhibitor, was determined by resuspending cells in serum-free conditional medium at a density of 20,000; 10,000; 5,000; or 2,000 cells/well in 6 – well low adherence plates (Corning, CA, USA). All experiments were conducted in triplicate. Plates were maintained at 37°C in humidified CO_2_ incubator and were fed every other day. After 10 days colonies were counted under the microscope in 10 fields per well.

### Cytotoxic Drug Treatment

Cells were treated with either doxorubicin (Pharmacia/Pfizer, Sandwich, UK), meloxicam (Sigma-Aldrich, MO, USA) or mavacoxib (Trocoxil™, Zoetis, London, UK) within the indicated dose range. All drugs were dissolved in dimethyl sulfoxide, and diluted in media immediately before use. Vehicle controls were included in all experiments.

### Analysis of Cytotoxicity

Cells were seeded in quadruplet in opaque 96-well plates (Corning, CA, USA) at 500 cells per well. Serial dilutions of either doxorubicin or meloxicam were added to the appropriate cells the following day or as indicated. Dose-response curves were generated 72 hours after exposure. Cytotoxicity was measured using the CellTiterGlo® Luminescent Cell Viability Assay (Promega, Madison, USA), which quantifies the number of viable cells in culture based on quantification of ATP present. Luminescence was recorded by luminometor (Viktor3, PerkinElmer, Massachusetts, USA). Data was averaged and normalized against the average signal of untreated/vehicle control treated samples.

### Colony Formation Assay

Cells were trypsinised into single cells and seeded at 500 cells/10 cm plate. The cells were treated with the indicated dose of doxorubicin or meloxicam whilst in suspension. Plates were incubated at 37°C in humidified CO_2_ incubator until colonies were visible. Growth media was changed once a week. The colonies were fixed by incubating with ice-cold methanol for 5 minutes at room temperature. Colonies were stained with Giemsa stain (Invitrogen, Paisley, UK) according to the manufacturer's instruction. The total number of colonies was counted.

### Invasion assay

The cell invasion ability of isolated cells was determined using the QCM™ collagen-based cell invasion assay kit (Millipore, MA, USA) according to the manufacturer's instructions. Cells were seeded into the upper inserts at 1×10^5^ cells per insert in William's E Medium with GlutaMAX. Outer wells were filled with William's E Medium with GlutaMAX. Cells were incubated at 37°C with 5% CO_2_ for 48 hours. The non-invading cells were removed. Cells that migrated through the gel insert to the lower surface were stained and quantified by colorimetric measurement at 560 nm.

### Chick Embryo Chorioallantoic Membrane Assay

Fertilised ISABrown layer strain chicken eggs (Roslin Institute Poultry Unit) were incubated in a humidified rotary incubator at 37°C. On day 3, a small window was opened in the shell after removal of 2–3 ml of albumin, to detach the CAM from the shell and to disclose the underlying CAM vessels. The window was sealed with tape and incubation was continued until day 7. On day 7, Single cell suspensions of adherent cells and mammospheres were labelled with PKH26 (Sigma-Aldrich, MO, USA), a red fluorescent live cell membrane dye, according to manufacturers' instructions. Viable 10^5^ (n = 4) cells were suspended in a 1∶1 mixture of serum-free media∶matrigel, and 25 µL were inoculated directly onto the CAM. The embryos were resealed and incubated without turning. At day 12, tumour growth and location were determined.

### Protein detection

Cells were lysed in urea lysis buffer (7 M urea, 0.1 M DTT, 0.05% Triton X-100, 25 mM NaCl, 20 mM Hepes pH 7.5). Equal amounts of protein were separated by SDS polyacrylamide gel electrophoresis (SDS PAGE), transferred to Hybond-C nitrocellulose membrane (Amersham Pharmacia Biotech, Buckinghamshire, UK) and hybridised to an appropriate primary antibody and HRP-conjugated secondary antibody for subsequent detection by ECL. Antibodies against Fibronectin and β-catenin were purchased from BD Biosciences (Oxford, UK). Anti-Zeb1, Anti-vimentin and β-actin were purchased from Abcam (Cambridge, UK). Anti-COX-2 (C-20) was purchased from Santa Cruz biotechnology (Texas, USA). Secondary antibodies were HRP-conjugated rabbit anti-mouse IgG and swine anti-rabbit IgG, were obtained from DakoCytomation (Glostrup, Denmark).

### RNA extraction and reverse transcription PCR analysis

Total cellular RNA was extracted using RNeasy® kit (Qiagen, CA, USA) and RNA quality was determined by A_260_ measurement. Semi-quantitative RT-PCR analysis of mRNA expression of stem cell specific genes including *Oct4*, *Nanog* and *STAT3* was performed using HotStar *Taq* polymerase (Qiagen, CA, USA) and specific primers (Table 3.)

**Table 3:** Primer sequences for the amplification of RT-PCR products from canine cell lines.

Gene	Forward primer (5′-3′)	Reverse Primer (5′-3′)	Product size
*Oct4*	CTCTGCAGCCAATCAACCACAA	GGAGAGGGGGATGAGAAGTACAAT	237 bp
*Nanog*	CTATAGAGGAGAGCACAGTGAAG	GTTCGGATCTACTTTAGAGTGAGG	160 bp
*STAT3*	GTGGAGAAGGACATCAGCGGTAA	AACTTGGTCTTCAGGTATGGGGC	250 bp
*β-Actin*	CATGTTTGAGACCTTCAACACCC	GCCATCTCTTGCTCGAAGTCCAG	229 bp

### Qualitative real-time PCR

Total RNA was reverse transcribed using the omniscript RT Kit (Qiagen, CA, USA) according to the manufacturer's instruction. Real-time PCR was performed on 50 ng of amplified RNA using a Stratagene M×3000p qPCR system (Aligent, CA, USA), using the Platinum® SYBR® Green qPCR SuperMix-UDG according to manufacturer's instruction (Invitrogen, CA, USA). Relative gene expression levels were obtained by normalization to the expression levels of housekeeping genes (*B2MG*, *RPL8*).

### Gene expression profiling using cDNA microarrays

RNA was isolated from frozen cell pellets of KTOSA5 spheres, adherent cells and MSCs with TriReagent (Sigma-Aldrich, MO, USA) according to manufacturer's instruction. Four independent replicates were used for each cell type. Total RNA quality was determined by Bioanalyser (Agilent, CA, USA) before further manipulation. Complementary RNA preparation and hybridization were performed by ARK-Genomics (Edinburgh, UK) using Affymetrix GeneChip® Canine Genome 2.0 Array (42,800 probe sets). Basic data analysis was performed using the Partek Genomics Suite (Partek Inc, MO, USA). Pathway analysis was performed using Ingenuity Pathway Analysis (IPA, Ingenuity systems; https://www.analysis.ingenuity.com). Genes from the dataset that met the log ratio cut-off of 1.5 were considered for the analysis. To identify the most relevant canonical pathways, we selected those that were statistically significant with a *p* value<0.005. All microarray data has been submitted to the NCBI Gene Expression Omnibus database (accession number GSE52063).

### Statistical analysis

Data were expressed as a mean + SD. Statistical analysis was performed with Minitab® statistical software (PA, USA) using analysis of variance and student's t test or mann-whitney test. The criterion for significance was p<0.05 for all comparisons.
